# Cannabinoid receptor 1 blockade protects human retinal pigment epithelial cells from oxidative injury

**Published:** 2013-02-13

**Authors:** Yan Wei, Xu Wang, Feng Zhao, Pei-quan Zhao, Xiao-li Kang

**Affiliations:** 1 Department of Ophthalmology, Xinhua Hospital, Shanghai Jiao Tong University School of Medicine, Shanghai, People's Republic of China; 2 Department of Oral and Maxillofacial-Head and Neck Oncology, College of Stomatology, Ninth People’s Hospital, Shanghai Jiao Tong University School of Medicine, Shanghai, People's Republic of China; 3 Department of Ophthalmology, Shuguang Hospital, Shanghai Traditional Chinese Medicine University, Shanghai, People's Republic of China

## Abstract

**Background:**

Because oxidative stress is assumed to be a key mechanism in the pathological process of age-related macular degeneration (AMD), increasing numbers of studies have focused on discovering new pathways and treatments for reducing oxidative damage. Our work investigates the potential role of the cannabinoid receptor 1 (CB1) in oxidative stress of primary human retinal pigment epithelial (RPE) cells, a cellular model of AMD.

**Methods:**

Primary human RPE cells were cultured and exposed to hydrogen peroxide for 24 h to induce oxidative damage. The expression of and changes in the CB1 receptor were determined with western blot assay and confocal imaging. The CB1 receptor in the RPE cells was inhibited with small interfering RNA (siRNA) or rimonabant (SR141716). Cell viability, apoptosis, and reactive oxygen species production were measured by using 3-(4,5-dimethylthiazol-2-yl)-2,5-diphenyl tetrazolium bromide (MTT) and sulforhodamine B assay, annexin V and propidium iodide staining, and the dichlorofluorescein fluorescence assay, respectively. Intracellular superoxide dismutase activity was assayed with a commercially available assay kit. Phosphoinositide 3-kinase/protein kinase B (PI3K/Akt) protein expression and activation of signaling molecules were assessed with western blot analysis.

**Results:**

We showed that human RPE cells express the CB1 receptor. In addition, oxidative stress upregulates the expression of the CB1 receptor. Deleting the CB1 receptor or treating with the CB1 receptor antagonist rimonabant (SR141716) rescued RPE cells from hydrogen peroxide–induced oxidative damage. Rimonabant pretreatment effectively reduced the apoptosis of RPE cells, inhibited the generation of intracellular reactive oxygen species and elevated the activity of superoxide dismutase. In addition, rimonabant significantly strengthened the oxidative stress-induced activation of the PI3K/Akt signaling pathway.

**Conclusions:**

The results demonstrate the expression and regulation of CB1 receptors in human RPE cells. Inhibiting the CB1 receptor may be an effective therapeutic strategy for AMD by downregulating oxidative stress signaling and facilitating PI3K/Akt activation.

## Introduction

Age-related macular degeneration (AMD) is a late-onset neurodegenerative retinal disease that shares many common clinical and pathological characteristics with other neurodegenerative disorders [1]. The characteristic features of AMD include degeneration, dysfunction, or loss of retinal pigment epithelial (RPE) cells caused by oxidative stress [2]. Therefore, treatments that target oxidative stress could be of great clinical significance for AMD.

The recently discovered endocannabinoid system (ECS), which consists of the endocannabinoids (the main cannabinoid 1 [CB_1_], cannabinoid 2 [CB_2_], and perhaps other yet not determined receptors) and their metabolizing enzymes (notably fatty acid amide hydrolase [FAAH]), has been implicated as an important instructive signal for controlling neuron survival in neurodegenerative disorders [3,4]. The ECS is also present in the human retina [5,6]. In addition to the protective effects against retinal toxicity [7], the ECS also regulates photoreception and neurotransmission in the optic nerve [8,9] and modulates the intraocular pressure and ocular blood vessels [10], suggesting an energetic role in ocular physiology. These beneficial effects of the ECS were thought to be mainly mediated by the CB_1_ receptor, the most abundant G-protein-coupled receptor in the central nervous system and the retina [11]. However, the pathophysiological functions of the CB_1_ receptor remain poorly understood in AMD.

In our previous study, we showed that the ARPE-19 cell line and primary human RPE cells express the CB_1_ and CB_2_ receptors and FAAH. Meanwhile, oxidative stress can upregulate CB_1_ and CB_2_ receptor expression and downregulate FAAH expression [12]. Other studies have also reported that endocannabinoid (anandamide, AEA) levels are elevated in the retina of patients with AMD [13]. Because the major effects of AEA are mediated by binding to the CB_1_ receptor, these findings raise the possibility of a direct effect of CB_1_ receptor signaling in the pathophysiological process of AMD. To assess the potential role of the CB_1_ receptor in the pathogenesis of RPE cell oxidative injury in AMD, we studied the status of CB_1_ receptors in the in vitro model of AMD. We next evaluated the effects of the selective CB_1_ receptor inhibitor, SR141716/rimonabant, or inhibition of the CB_1_ receptor by small interfering RNA (siRNA) in human primary RPE cells exposed to oxidative stress. Our study demonstrates that inhibiting the CB_1_ receptor attenuated retinal oxidative stress, decreased the generation of intracellular ROS, elevated the activity of superoxide dismutase (SOD), and strengthened oxidative stress-induced activation of the phosphoinositide 3-kinase/protein kinase B (PI3K/Akt) signal pathway. Our findings might set the basis for pharmacological modulation of the CB_1_ receptor as a novel therapeutic option for AMD.

## Methods

### Primary human retinal pigment epithelial cell culture

Human RPE cells were obtained from eye bank donor eyes. The eyes were cut across the posterior pole, and the vitreous and neural retinas were removed. The remaining eyecups were washed with phosphate buffered saline (PBS, 136.8 mM NaCl, 2.7 mM KCl,1.8 mM KH_2_PO_4_ and 4 mM Na_2_HPO_4_ in distilled water, pH 7.4) and incubated with 0.025% trypsin-EDTA (Invitrogen-Gibco, Carlsbad, CA) in a humidified chamber at 37 °C. The cells were then gently scraped and seeded in Dulbecco’s modified Eagle’s medium (DMEM; Gibco) containing 15% fetal bovine serum (FBS; Gibco) and were cultured at 37 °C in a humidified atmosphere of 95% air and 5% CO_2_. The medium was changed every 2 days. Human RPE cells were used within 10 generations and were quite sensitive to oxidative stress.

### 3-(4,5-dimethylthiazol-2-yl)-2,5-diphenyl tetrazolium bromide assay for cell viability

The 3-(4,5-dimethylthiazol-2-yl)-2,5-diphenyl tetrazolium bromide (MTT) assay is a qualitative index of cell viability. Mitochondrial and cytosolic dehydrogenases of living cells reduce the yellow tetrazolium salt (MTT) to produce a purple formazan dye that can be detected spectrophotometrically [14]. The RPE cells were seeded in a flat-bottomed microculture 96-well plate (1.5×10^4^ cells/well) and allowed to adhere for 24 h. Cells at approximately 70%–80% confluence were treated with various concentrations of H_2_O_2_ in serum-free and phenol-free DMEM/F12 medium for 24 h. Dose–response assays were performed on RPE cells to determine the half maximal inhibitory concentration (IC_50_) of hydrogen peroxide (H_2_O_2_). The 30% H_2_O_2_ stock solution was used within 3 months. Working solutions of H_2_O_2_ were freshly made and added to serum-free, phenol red-free DMEM/F12 medium. Rimonabant (SR141716A), a selective CB_1_ receptor antagonist (NIDA Drug Supply, Research Triangle Park, NC), was dissolved in DMSO. ACEA (Tocris Bioscience, Ellisville, MO), a selective CB_1_ receptor agonist, was dissolved in anhydrous ethanol. RPE cells were preincubated with various concentrations of rimonabant and/or ACEA for 15 min before being exposed to 200 μM H_2_O_2_ for 24 h in serum-free, phenol red-free DMEM/F12 media at 37 °C. For each concentration of H_2_O_2_ and compounds, five wells were analyzed. Each experiment was performed at least three times.

After the treatment described above, MTT (Sigma, St. Louis, MO) was added to a final concentration of 0.5 mg/ml and incubated for 4 h at 37 °C. The culture medium was then removed, and the remaining blue precipitate was solubilized in DMSO followed by an absorbance reading at 570 nm in a plate reader using 630 nm as a reference (Spectra Max 340; Molecular Devices, Sunnyvale, CA). This reading was divided by the adjusted absorbance reading of untreated cells in control wells to obtain the percentage of cell survival.

### Sulforhodamine B cell proliferation assay

The sulforhodamine B (SRB) assay is used for determining cell viability, based on measuring cellular protein content. The RPE cells were seeded in microculture 96-well plates at a cell density of 1.5×10^4^ cells/well and allowed to adhere for 24 h. On the following day, the RPE cells were preincubated with various concentrations of rimonabant for 15 min before being exposed to 200 μM H_2_O_2_ for 24 h in serum-free, phenol red-free DMEM/F12 media at 37 °C. After the incubation period, the media were removed, and the cells were fixed with 10% (W/V) trichloroacetic acid for 10 min, and then stained for 30 min with SRB dissolved in 1.0% acetic acid, after which the excess dye was removed by washing repeatedly with 1.0% acetic acid. The protein-bound dye was dissolved in 10 mM Tris base solution to determine optical density (OD) at 510 nm using a microplate reader.

### RNA interference

CB_1_ receptor siRNA and the negative control siRNA were obtained from Gene Pharma, Shanghai, China. The expression of the CB_1_ receptor was reduced using previously reported target-specific siRNA molecules. The primers for the CB_1_ receptor (si-CB_1_) were as follows: sense 5′-GAG CAU GUU UCC CUC UUG UTT-3′; antisense 5′-ACA AGA GGG AAA CAU GCU CTT-3′. The primers for the negative control [15] (si-NC) were as follows: sense 5′-UUC UCC GAA CGU GUC ACG UTT-3′; antisense 5′-ACG UGA CAC GUU CGG AGA ATT-3′. Target or control siRNA was transfected into cells at 40%–60% confluence using the Lipofectamine 2000 reagent (Invitrogen, New York, NY) according to the manufacturer’s instructions.

### RNA extraction and real-time reverse transcription polymerase chain reaction

Total RNA was isolated from primary human RPE cells and H_2_O_2_ (0–300 μM)-treated RPE cells using the RNeasy Total RNA System (RNeasy Mini Kit, Qiagen, Valencia, CA) following the manufacturer's recommendation and then treated with RNase-free DNase I to remove any contaminating genomic DNA. The isolated RNA had OD 260/280 ratios greater than or equal to 2.0. To synthesize cDNA templates for PCR, 1 μg of total RNA was reverse transcribed with oligo-(dT) primer and reverse transcriptase (ReverTra Ace, Toyobo Co., Ltd., Osaka, Japan). The quality of the first-strand cDNA was confirmed by PCR with β-actin primers.

Real-time reverse transcription polymerase chain reaction (RT–PCR) was performed for quantitative analysis according to the standard protocol using the SYBR Green PCR Master Mix (Toyobo Co., Ltd., Osaka, Japan). The PCR conditions for the CB_1_ receptor were as follows: after initial denaturation at 95 °C for 5 min, 40 cycles of 94 °C for 30 s, 58 °C for 30 s, and 72 °C for 1 min were performed, followed by a 10 min extension at 72 °C. The primers used were as follows: for the CB_1_ receptor, 5′-TTC CCT CTT GTG AAG GCA CTG-3′ (sense) and 5′-TCT TGA CCG TGC TCT TGA TG C-3′ (antisense) [16]; and for β-actin, 5′-GAT GAG ATT GGC ATG GCT TT-3′ (forward) and 5′-GAG AA G TGG GGT GGC TT-3′ (reverse) [12]. Quantification of CB_1_ receptor mRNA was normalized with β-actin as the reference. The specificity of the PCR amplification products was checked by performing a dissociation melting curve analysis. Relative multiples of changes in mRNA expression were determined with the relative comparative threshold method [17].

### Western blot analysis

RPE cells were plated into six-well plates (1.5×10^5^ cells/well). To evaluate the expression of the CB_1_ receptor, the cells were treated with H_2_O_2_ (0–300 μM) in serum-free and phenol-free DMEM/F12 medium for 24 h. To determine the expression of PI3K/Akt, the cells were pretreated with or without rimonabant (0.1, 1 μM) for 15 min and then exposed to H_2_O_2_ (200 μM) for 24 h. After the treatment, the cells were rinsed twice with ice-cold PBS, then scraped into cell lysis buffer, and centrifuged at 12,314 × *g* for 10 min at 4 °C. Protein levels were determined using the bicinchoninic acid (BCA) protein assay (Pierce, Rockford, IL). Fifteen micrograms of total protein was solubilized in 2% sodium dodecyl sulfate sample buffer, separated on a 10% sodium dodecyl sulfate–PAGE and transferred to nitrocellulose membranes by electroblot. Blots were washed in Tris-buffered saline containing 0.1% Tween-20 and 5% nonfat dairy milk, and incubated in antibodies to the CB_1_ receptor (rabbit polyclonal 1:1000; Abcam, Cambridge, UK), PI3K/Akt, and glyceraldehyde 3-phosphate dehydrogenase (mouse monoclonal 1:10,000; Cell Signaling Technology, Danvers, MA) at 4 °C overnight. Blots were washed three times, incubated with horseradish peroxidase–conjugated goat anti-rabbit immunoglobulin G (1:3000; Pierce) or horseradish peroxidase–conjugated goat anti-mouse immunoglobulin G (1:20,000; Pierce) and developed using chemiluminescence (SuperSignal West Pico Luminescent; Pierce) according to the manufacturer’s instructions.

### Immunofluorescent staining

CB_1_ receptor expression in RPE cells was determined with immunofluorescence staining. Briefly, RPE cells were grown to confluence in chamber slides (Nalgene-Nunc, Lab-Tek, New York, NY). Cells were incubated with or without 200 μM H_2_O_2_ for 24 h at 37 °C. The growth medium was aspirated, and the cells were washed three times with PBS and then fixed with 4.0% paraformaldehyde for 20 min at 4 °C. After the cells had been washed with PBS, they were permeabilized with 0.2% Triton X-100 in PBS for 15 min at room temperature. Subsequently, CB_1_ receptor expression in RPE cells was determined with immunofluorescence staining using anti-CB_1_ (rabbit polyclonal, Abcam), at a 1:100 dilution for 6 h at 4 °C. After the cells had been rinsed with PBS, they were probed with goat anti-rabbit fluorescein isothiocyanate (FITC; 1:250; Pierce) for 1 h at room temperature. Nuclei were counterstained with 4’,6-diamidino-2-phenylindole (Molecular Probes). The slides were washed and photographed with a laser scanning confocal microscope (TCS SP2, Leica, Wetzlar, Germany) [18].

### Apoptosis assay with annexin V/propidium iodide staining

The apoptosis rate of the RPE cells was evaluated using the annexin V/propidium iodide (PI) double staining assay. Annexin V binds to phosphatidylserine exposed on the cell membrane, one of the earliest indicators of cellular apoptosis. Using a viability dye such as PI allows early apoptotic, late apoptotic, and necrotic cells to be distinguished. RPE cells were preincubated with various concentrations of rimonabant for 15 min before being exposed to H_2_O_2_ (200 μM) for 24 h in serum-free, phenol red-free DMEM/F12 media at 37 °C. The apoptosis rate of the RPE cells was evaluated using an Annexin V-FITC Apoptosis Detection Kit (Invitrogen) and determined with flow cytometry. Staining procedures were performed according to the manufacturer's instructions. Using the various labeling patterns in this assay, the following cell populations were identified: normal (PI^−^/annexin V^−^), early apoptotic (PI^−^/annexin V^+^), and cells undergoing apoptosis/necrosis (PI^+^/annexin V^+^).

### Reactive oxygen species determination

The intracellular level of ROS is an important biomarker for oxidative stress: Increased ROS levels generally indicate increased oxidative stress. Relative ROS production was determined by the formation of a fluorescent dichlorofluorescein (DCF) compound upon the oxidation of the non-fluorescent, reduced DCF-DA [19]. RPE cells were preincubated with various concentrations of rimonabant for 15 min before being exposed to H_2_O_2_ (200 μM) for 30 min in serum-free, phenol red-free DMEM/F12 media at 37 °C. After the treatment, the cells were incubated with 10 μM DCF-DA at 37 °C for 30 min and then washed twice with PBS. Relative fluorescence was measured using a fluorescence plate reader at 485 nm excitation and 535 nm emission wavelengths (Wallac; Perkin-Elmer, Watham, MA).

### Superoxide dismutase measurement

The intracellular SOD activity was assayed with a commercially available assay kit (Jiancheng Biochemical Inc., Nanjing, China) using a xanthine and xanthine oxidase system to produce superoxide. The RPE cells were pretreated with or without rimonabant (1 μM) for 15 min and then exposed to H_2_O_2_ (200 μM) for 24 h. The superoxide oxidized hydroxylamine to nitrite to form a carmine color agent. The optical density at 550 nm was measured with a microplate reader.

### Statistical analysis

The data are presented as the mean ± standard error of the mean (SEM) of the results of two or three separate experiments, as specified in the figure legends. The data were analyzed using ANOVA (ANOVA) or a Student *t* test with the SPSS software (SPSS, Beijing, China), and a p value <0.05 was considered significant.

## Results

### Expression of and changes in cannabinoid receptor 1 in retinal pigment epithelial cells

**Figure 1:**
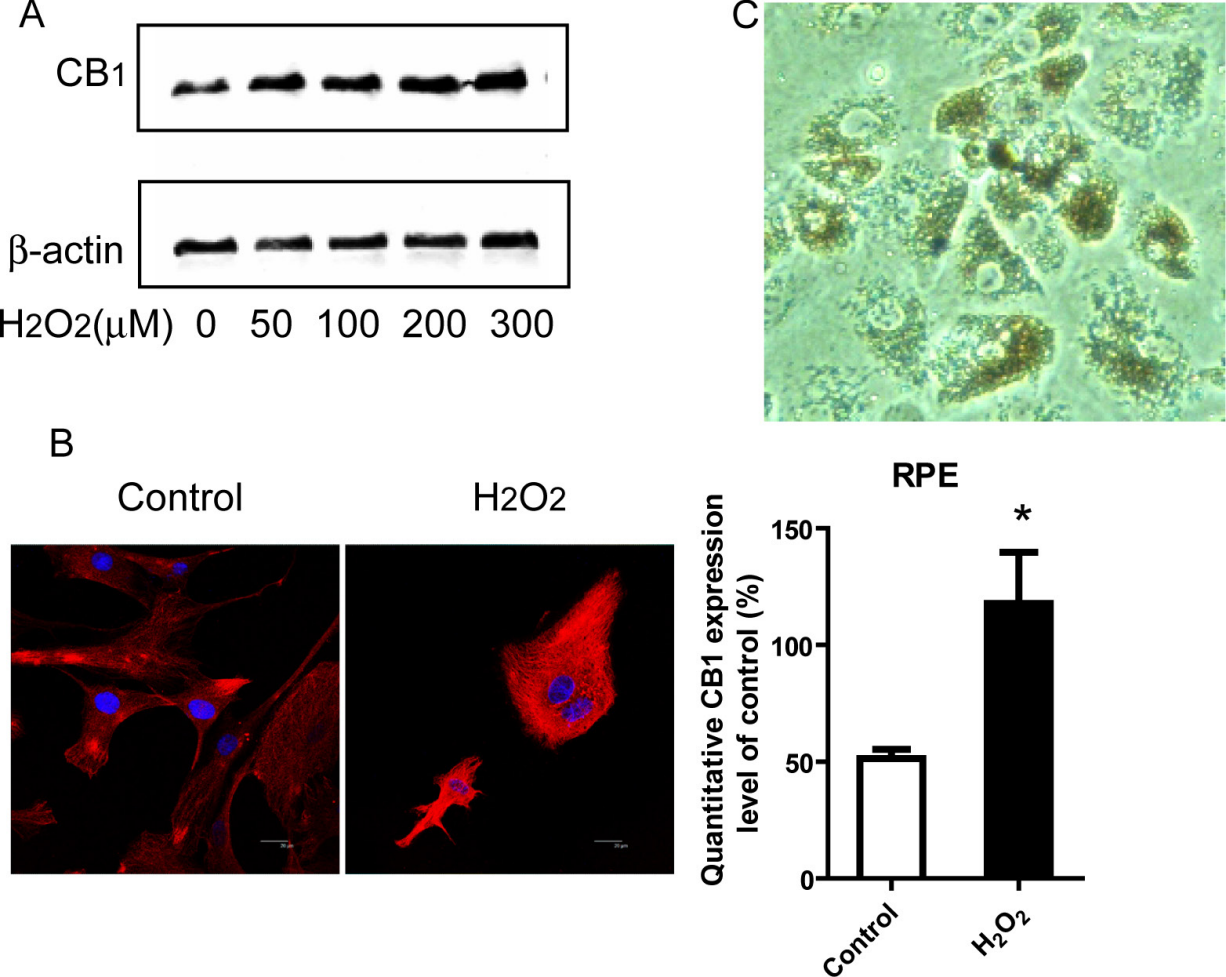
Expression of and changes in the CB_1_ receptor in human retinal pigment epithelial (RPE) cells. **A**: In the western blot analysis of CB_1_ receptor protein expression changes in primary RPE cells, CB_1_ receptor protein level was significantly increased by H_2_O_2_ incubation in a dose-dependent manner. **B**: CB_1_ receptor protein localized to the cytoplasm and cellular membrane as demonstrated with immunofluorescence staining (Bar=20 μm). Quantitative analysis of the fluorescent levels is indicated in the right panel. *p<0.05 versus control, the sample number is n=5 per group and we performed *t* test here. **C**: A representative photograph of primary cultured RPE cells seeded for 24 h.

RPE cells were treated with H_2_O_2_ (0–300 μM) for 24 h, and the changes in CB_1_ receptor protein expression were determined with western blot assay. The results show that the CB_1_ receptor protein was significantly increased by H_2_O_2_ incubation in a dose-dependent manner in human primary RPE cells (Figure 1A). Similar results were obtained with immunofluorescence assays. By using immunofluorescence assays and images visualized with a confocal platform, we also detected that the red fluorescence of the CB_1_ receptor was upregulated by H_2_O_2_ incubation (Figure 1B). A representative photograph of the primary cultured RPE cells seeded for 24 h is shown in Figure 1C. These data suggest that the CB_1_ receptor is localized in the primary RPE cells and is induced by H_2_O_2_ incubation.

### RNA interference against cannabinoid receptor 1 rescued retinal pigment epithelial cells from hydrogen peroxide–induced cellular damage

**Figure 2:**
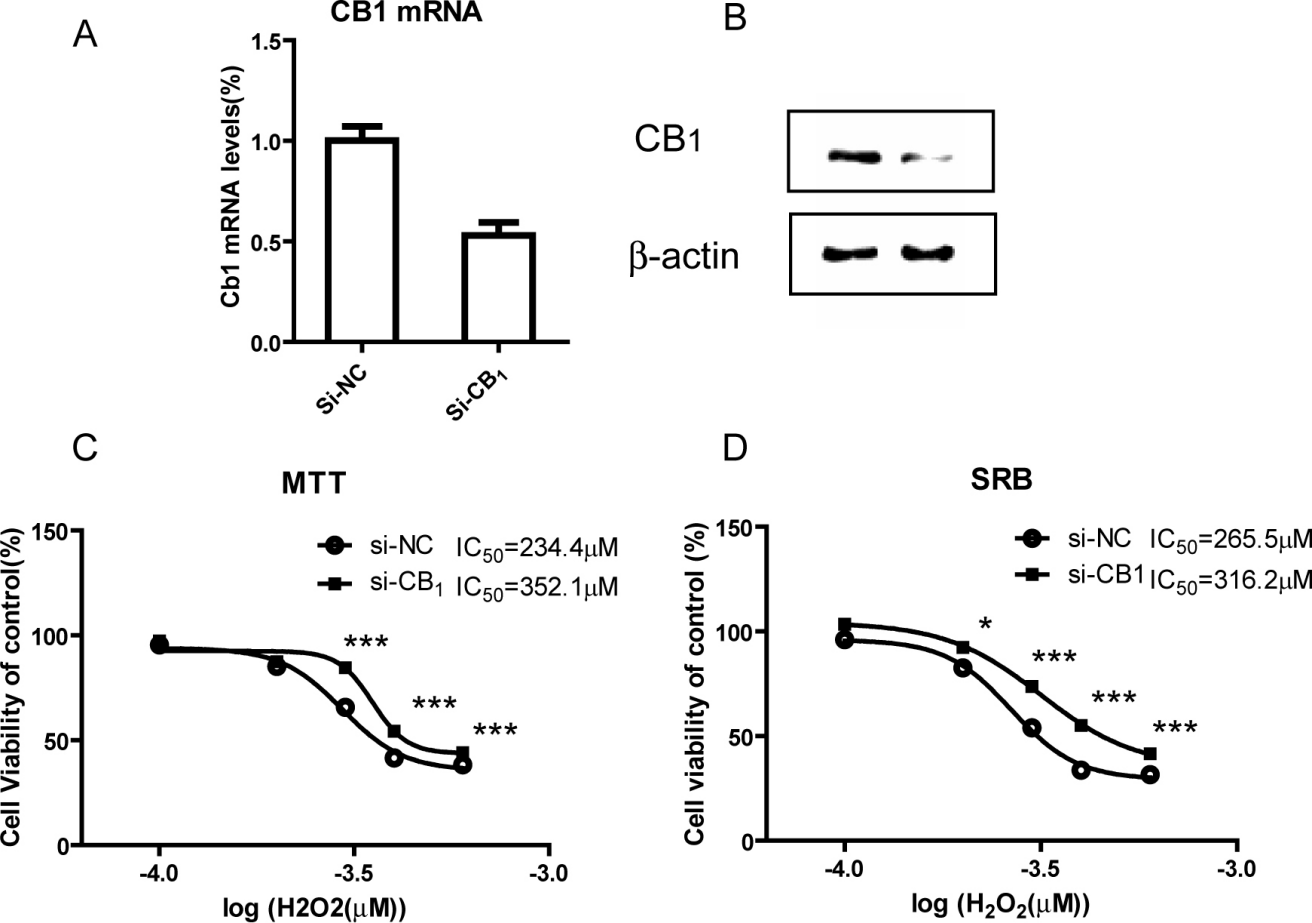
Downregulation of the CB_1_ receptor protected RPE cells from H_2_O_2_-induced damage. **A**: Primary human RPE cells were transfected with CB_1_ receptor siRNA for 24 h. CB_1_ receptor mRNA levels were detected using real time RT–PCR. **B**: Primary human RPE cells were transfected with CB_1_ receptor siRNA for 48 h, and CB_1_ receptor protein levels were detected by western blot assay. **C**: After the si-NC or si-CB_1_ siRNA-treated RPE cells received different concentrations of H_2_O_2_ for 24 h, cell viability was examined with the MTT assay. ***p<0.001, si-NC versus si-CB_1_ siRNA-treated cells. **D**: After the si-NC or si-CB_1_ siRNA-treated RPE cells received different concentrations of H_2_O_2_ for 24 h, cell viability was examined with the SRB assay. *p<0.05,***p<0.001, si-NC versus si-CB_1_ siRNA-treated cells. The statistical test of **C** and **D** are two way ANOVA, n=4 per group.

To examine if negative regulation of the CB_1_ receptor contributes to protecting RPE cells from H_2_O_2_-induced oxidative stress, we used CB_1_ receptor-specific siRNA to reduce the CB_1_ receptor mRNA and protein expression in RPE cells. The RNA interference efficiency was determined with real-time RT–PCR and western blot analysis, and the mRNA and protein levels of the CB_1_ receptor were significantly reduced in RPE cells after treatment with 50 pM CB_1_ receptor siRNA for 48 h (Figure 2A,B). The MTT and SRB assay for cell viability was used to quantify the cytotoxic response of the RPE cells. In cells with lower CB_1_ receptor expression, H_2_O_2_ caused reduced damage to cell viability than the negative control sequence-treated cells (Figure 2C,D). These data suggest that inhibiting the CB_1_ receptor could rescue RPE cells from oxidative damage.

### The cannabinoid receptor 1 antagonist rimonabant rescued retinal pigment epithelial cells from oxidative damage

**Figure 3:**
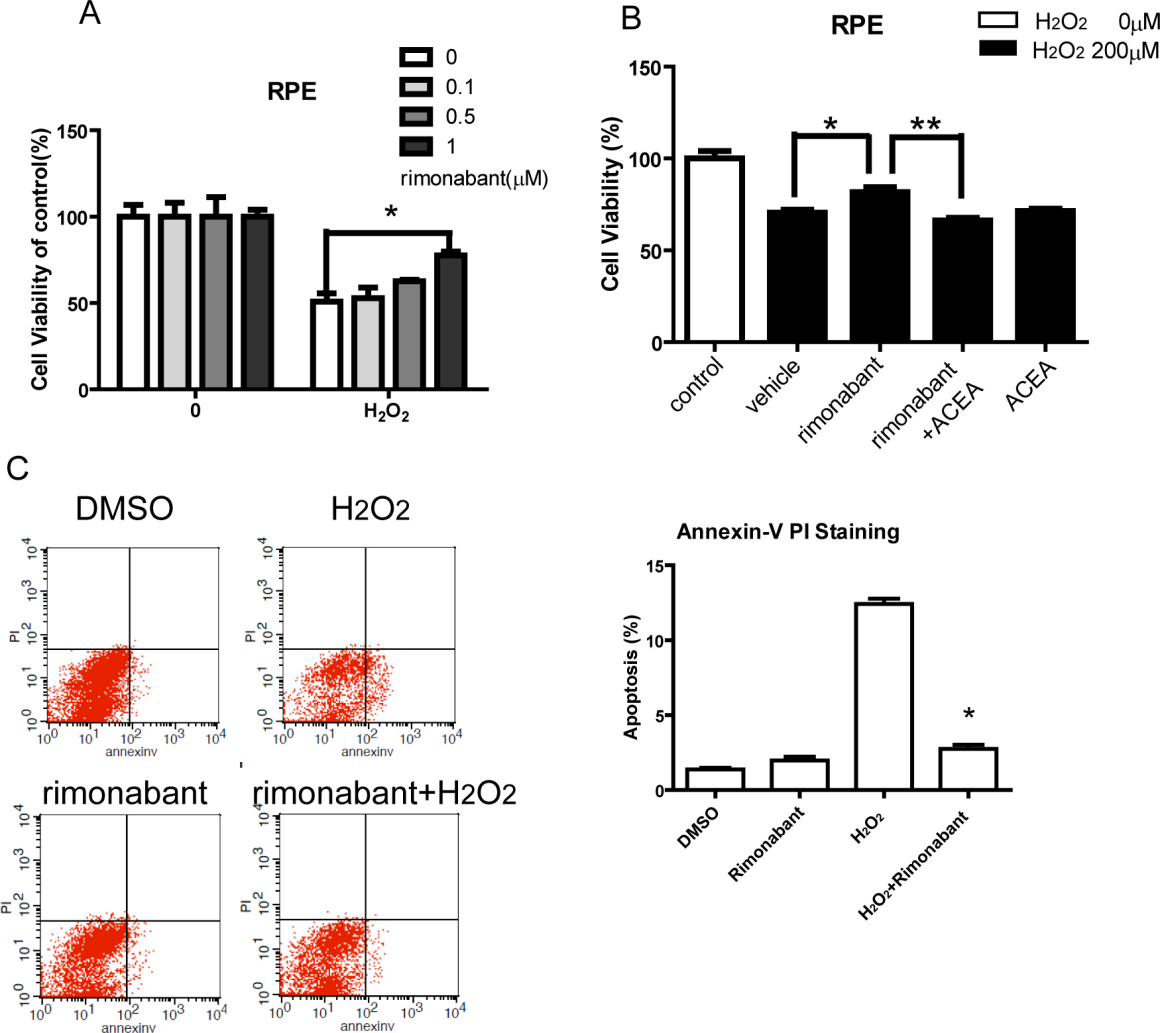
Rimonabant attenuates H_2_O_2_-induced cytotoxicity and apoptosis. **A**: Inhibition of the H_2_O_2_-induced decrease in RPE cell viability by rimonabant. RPE cells were pretreated with rimonabant (0 to 5 μM) for 15 min before being exposed to H_2_O_2_ (200 μM) for 24 h, and cell viability was measured with the MTT assay. Values are the percentage of control (no H_2_O_2_, no rimonabant). *p<0.05 versus H_2_O_2_. **B**: RPE cells were pretreated with 1 μM ACEA for 15 min in the presence or absence of rimonabant (1 μM) before being exposed to H_2_O_2_ (200 μM) for 24 h. *p<0.05 versus H_2_O_2_. **p<0.01 versus rimonabant without ACEA. **C**: Flow cytometric analysis of cell death with DMSO, H_2_O_2_ (200 μM), rimonabant (1 μM), and rimonabant (1 μM) + H_2_O_2_ (200 μM). Cells were treated with different media as indicated for 24 h. Summary of the results showing a significant increase in apoptosis in RPE cells maintained in H_2_O_2_ (200 μM) compared with those maintained in vehicle. When the cells were incubated with rimonabant (1 μM), H_2_O_2_-induced apoptosis was significantly reduced. Treatment of RPE cells with rimonabant (1 μM) alone did not alter cell death. *p<0.05 versus vehicle H_2_O_2_ (n=4). The statistical test of **A** and **B** are one way ANOVA, n=4 per group. In the **C**, the test is two way ANOVA, n=4 per group.

We selected rimonabant, a potent selective CB_1_ receptor antagonist, to pharmacologically inhibit the CB_1_ receptor. RPE cells were treated with H_2_O_2_ for 24 h to induce a dose-dependent decrease in cell viability, with an IC_50_ value of 234.4 μM. Pretreatment of RPE cells with rimonabant for 15 min significantly protected against H_2_O_2_-induced toxicity at concentrations of 1 μM to 86.2% of the control (Figure 3A). RPE cells treated with 0, 0.1, 0.5, and 1 μM rimonabant alone showed no significant difference in viability compared to the untreated control cells. Pretreatment with 1 μM ACEA (a potent selective agonist of the CB_1_ receptor) in the presence of 1 μM rimonabant significantly decreased the cytoprotective effect of rimonabant. Pretreatment with ACEA alone did not show any protection against H_2_O_2_-induced cell death (Figure 3B). In addition, RPE cells maintained in H_2_O_2_ showed a significant increase in apoptosis as indicated by annexin V/ PI using flow cytometry; this increase was attenuated with rimonabant (1 μM; Figure 3C). These data suggest that the pharmacological inhibition of the CB_1_ receptor also protected RPE cells from H_2_O_2_-induced damage.

### Cannabinoid receptor 1 inhibition attenuates hydrogen peroxide–induced intracellular reactive oxygen species production and increases intracellular superoxide dismutase activity

**Figure 4:**
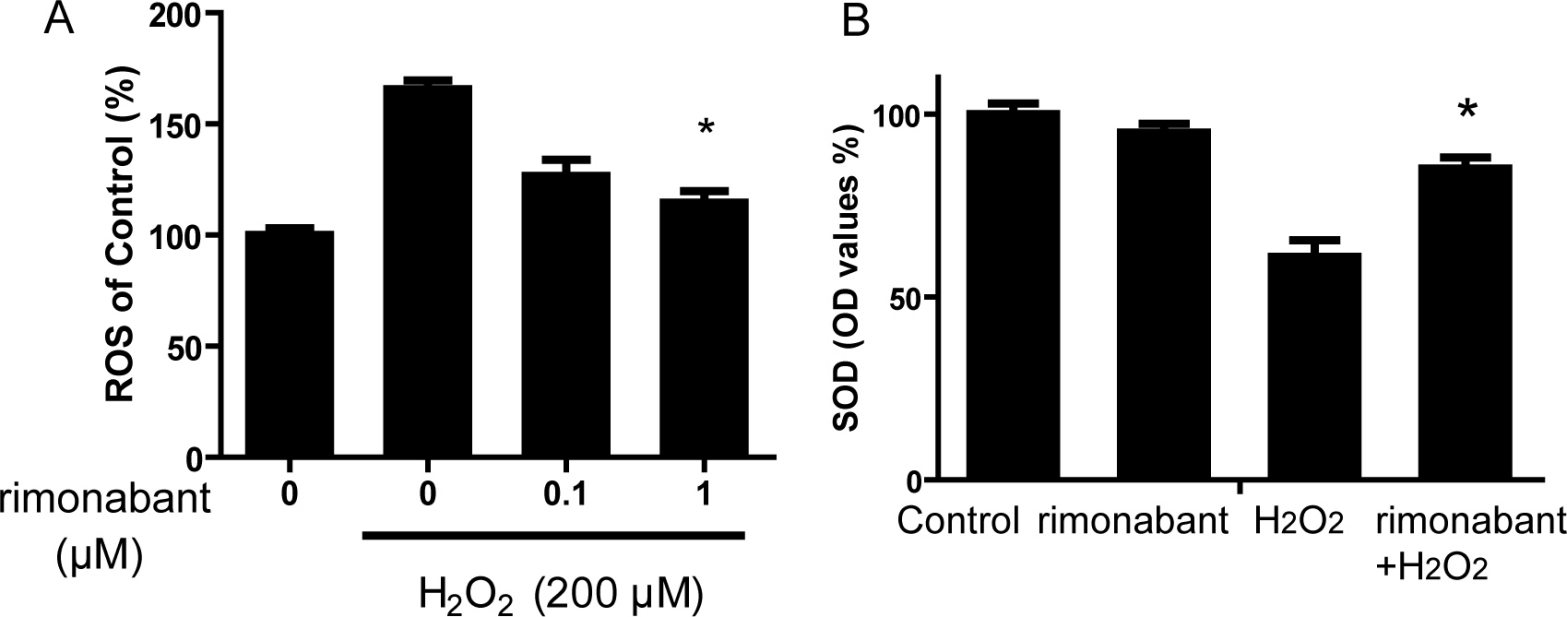
Rimonabant inhibited the H_2_O_2_-induced increase in intracellular ROS and activated the H_2_O_2_-induced decrease in intracellular superoxide dismutase (SOD) in RPE cells. **A**: RPE cells were pretreated with rimonabant (0 to 1 μM) for 15 min before being exposed to H_2_O_2_ (200 μM) for 24 h. Intracellular ROS was measured with the DCF-DA assay. *p<0.05 versus H_2_O_2_. **B**: SOD activity was assayed with a commercially available assay kit. *p<0.05 versus control (no H_2_O_2_ no rimonabant). #p<0.05 versus H_2_O_2_. The statistical test of **A** is one way ANOVA, n=4 per group. In the **B**, the test is two way ANOVA, n=4 per group.

To explore the possible mechanism of the protective effects of the CB_1_ receptor blockade, we next examined its effects on H_2_O_2_-induced oxidative stress in RPE cells. As shown in Figure 4A, treatment with 200 μM H_2_O_2_ for 30 min induced a significant increase in intracellular ROS formation: approximately 1.7 times as indicated with DCF fluorescence compared with controls, whereas pretreatment with 1 μM rimonabant for 15 min significantly reduced ROS generation.

As SOD is the major cellular anti-ROS agent, we also measured SOD activity following rimonabant incubation. Treatment with 200 μM H_2_O_2_ for 24 h caused an obvious decrease (33%) in the total intracellular SOD activity in RPE cells, and rimonabant pretreatment at 1 μM for 15 min significantly prevented a decrease in SOD activity. Treatment with rimonabant alone did not affect SOD activity (Figure 4B). These data suggest that rimonabant could activate the cellular antioxidative system to protect RPE cells.

### Rimonabant enhances the hydrogen peroxide–induced activation of phosphoinositide 3-kinase/protein kinase B

**Figure 5:**
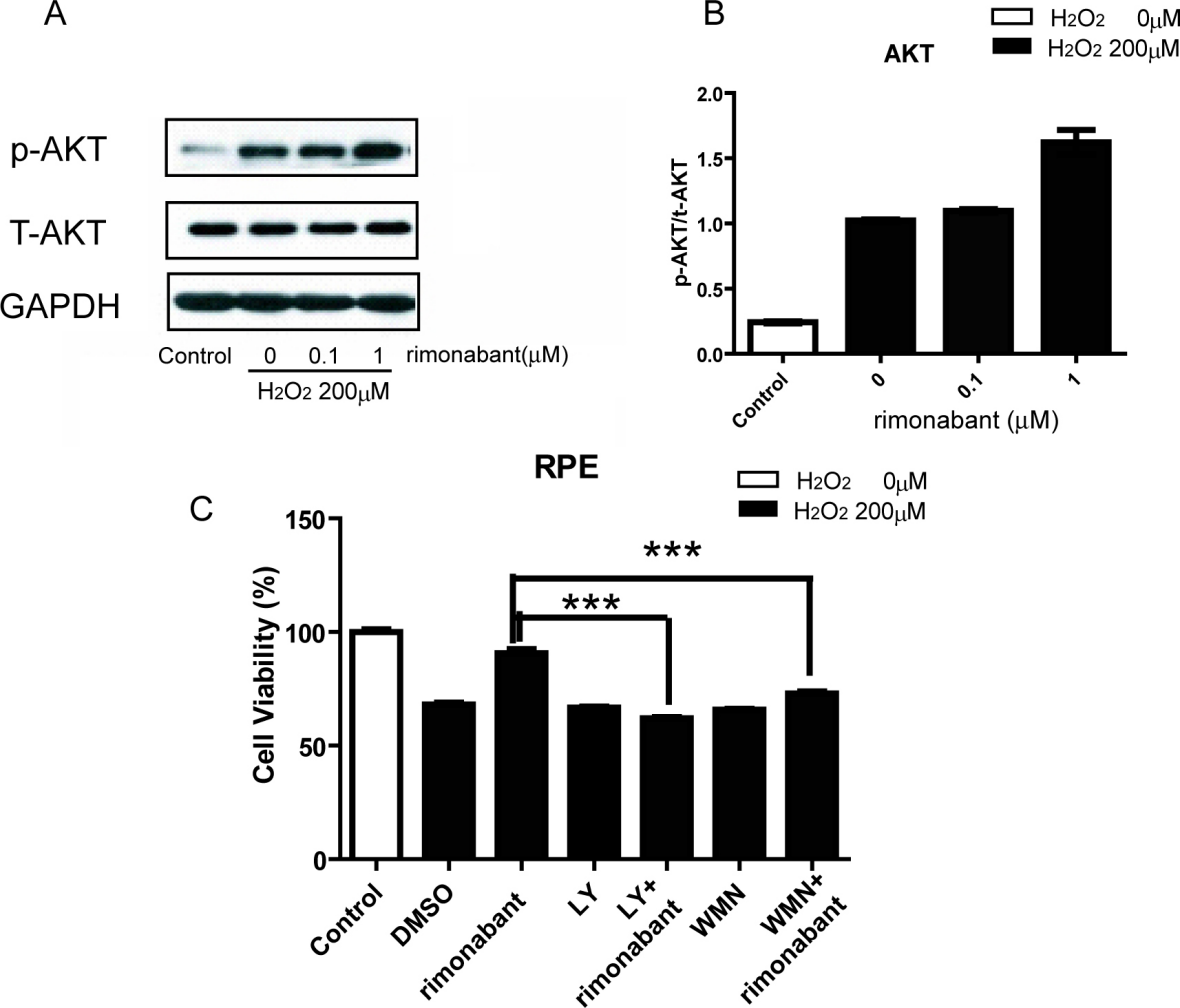
Rimonabant modulates phosphorylation of the PI3K/Akt signaling pathway. **A**: Representative western blot analysis shows that rimonabant (1 μM) enhanced the H_2_O_2_-induced activation of p-PI3K/Akt in RPE cells. RPE cells were pretreated with rimonabant (0.1, 1 μM, 15 min) and then exposed to H_2_O_2_ (200 μM, 24 h). **B**: Statistical analysis of the results indicated a 2.4-fold increase. **C**: RPE cells were pretreated with or without LY294002 (LY, 10 μM) or wortmannin (WMN, 10 μM) for 15 min in the presence or absence of rimonabant (1 μM) before being exposed to H_2_O_2_ (200 μM) for 24 h (***p<0.001 versus rimonabant + H_2_O_2_). The statistical test of **C** is one way ANOVA, n=4 per group.

To address the potential role of PI3K/Akt in mediating the rimonabant protection of RPE cells from oxidative injury, phosphorylation of PI3K/Akt was assessed with western blot analysis. The results show that PI3K/Akt is activated by H_2_O_2_. Pretreating RPE cells with 1 μM rimonabant followed by 200 μM H_2_O_2_ enhanced PI3K/Akt activity compared to cells treated with H_2_O_2_ alone (Figure 5A). We further introduced two specific inhibitors of PI3K/Akt, LY294002 and wortmannin, to block PI3K/Akt activation. RPE cells were pretreated with 10 μM LY294002 or wortmannin for 15 min in the presence or absence of rimonabant, followed by an H_2_O_2_ challenge for 24 h. As shown in Figure 5B, LY294002 and wortmannin abrogated the rimonabant protection of RPE cells from oxidative injury.

## Discussion

The main findings of the current study are as follows: (1) The CB_1_ receptor not only is present in the primary cultured RPE cells but also is upregulated by H_2_O_2_-induced oxidative stress dose dependently. (2) Inhibiting the CB_1_ receptor with siRNA or rimonabant (SR141716) prevents H_2_O_2_ -induced RPE cell death. (3) Inhibiting the CB_1_ receptor ameliorates H_2_O_2_-induced RPE cell oxidative stress, reduces intracellular ROS production, increases cellular SOD activities, and enhances the phosphorylation of PI3K/Akt. Several preventative strategies are under consideration for AMD [20]. Because oxidative stress is believed to be an important mediator in the RPE cells dysfunction and contributes to the pathogenesis of AMD [21], current prophylactic treatments center on reducing or protecting RPE cells from oxidative damage. We used H_2_O_2_ to induce RPE cell damage in our experiments for several reasons. First, hydrogen peroxide (H_2_O_2_), a byproduct of oxidative stress, has been reported to trigger apoptosis in human RPE cells, and the initial loss of RPE cells in AMD may result from apoptosis [22]. Second, H_2_O_2_, a membrane-permeable oxidant, as one of the major radicals as well as a precursor of highly oxidizing, tissue-damaging radicals, can enter cells and induce cytotoxicity because of its high membrane permeability. Third, H_2_O_2_ has been found in ocular tissues in vivo [23] and can be produced by RPE cells as a reactive oxygen intermediate during photoreceptor outer segment phagocytosis [24]. Thus, H_2_O_2_ added to the culture medium was used as a chemical oxidant.

Recent studies have demonstrated that the CB_1_ receptor blockade ameliorates inflammation, oxidative stress, and cell death in models of neuronal injury [25-30]. However, a potential role of the CB_1_ receptor in the pathogenesis of AMD has not been previously explored. In this study, we found that the CB_1_ receptor not only is present in the primary cultured RPE cells but also is upregulated by H_2_O_2_-induced oxidative stress in the cellular model of AMD [12]. This result is also consistent with the elevated endocannabinoid anandamide (the endogenous ligand for CB_1_ receptors) levels observed in retinas of patients with AMD [31]. The finding proposed an interesting question about the possible role of the CB_1_ receptor signaling in RPE cell viability and further in the pathophysiological process of AMD. Cannabinoid receptors can be increased by signals provided by cells in the tissue microenvironment, such as inflammatory stimuli and cytokines [32]. We hypothesized that, as in other tissues and organs, changes in the levels of the CB_1_ receptor may be related to the pathogenesis and/or the on-demand adaptive changes of neuroinflammatory conditions of AMD [33]. Using inhibition of the CB_1_ receptor with siRNA or rimonabant (SR141716) in human primary RPE cells exposed to H_2_O_2_, a cellular model of AMD, we further explored the role of the CB_1_ receptor in the pathogenesis of AMD. We demonstrated that CB_1_ receptor-specific siRNA rescued RPE cells from oxidative stress. This phenomenon increases the possibility that the CB_1_ receptor may become a treatment target for AMD. We also introduced rimonabant, a selective CB_1_ receptor antagonist, in oxidative stress-induced RPE cellular damage. We found that rimonabant protected RPE cells from oxidative stress-induced cell damage and intracellular ROS generation in a dose-dependent manner with high efficacy. We further tested whether rimonabant exerted its protective role via CB_1_ receptor inhibition. Coincubation with ACEA, a specific agonist of the CB_1_ receptor, abrogated the rimonabant protection of RPE cells from oxidative injury, suggesting that rimonabant exerts its protective effect via CB_1_ receptor activity. We also explored whether rimonabant induced survival signals while rescuing RPE cells from oxidative damage. Activation of the PI3K/Akt pathway–mediated antioxidant defense had been suggested to protect RPE cells from oxidative stress [34-36]. We therefore assessed whether rimonabant induced modification of the PI3K/Akt pathway in oxidative injury, and we found that rimonabant significantly extended the H_2_O_2_-induced activation of the PI3K/Akt pathway.

**Figure 6:**
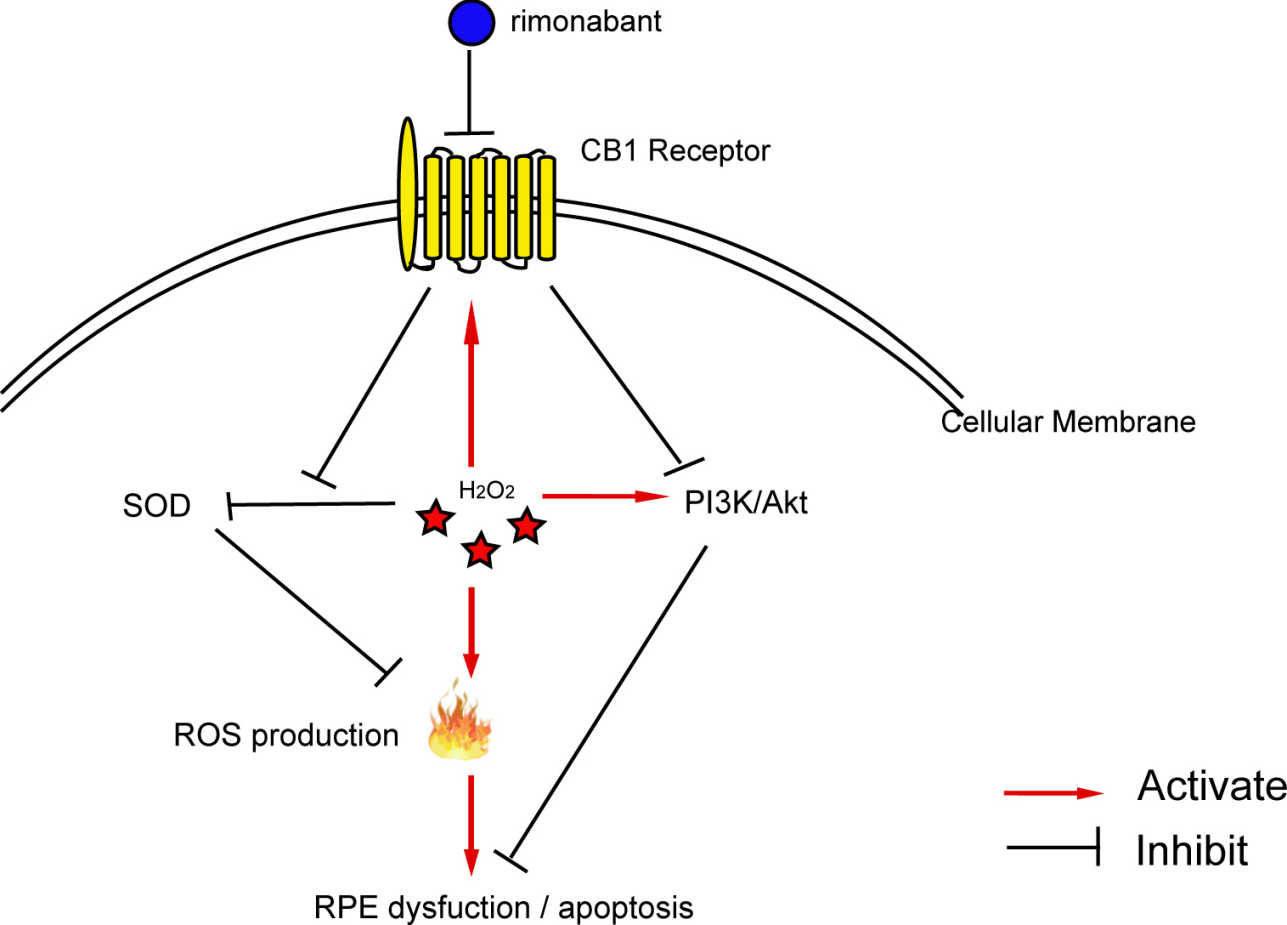
The mechanism chart of CB_1_ receptor blockade protects human RPE cells from H_2_O_2_-induced damage.

In summary, our results demonstrate that expression of the CB_1_ receptor was significantly increased in the cellular model and that pharmacological blockade and/or inhibition of the CB_1_ receptor with siRNA ameliorated H_2_O_2_-induced retinal oxidative stress and production of SOD, and prevented cell death. RPE cells perform vital functions for safeguarding photoreceptor cells against oxidative stress and are involved in the pathogenesis of AMD (Hypothesis model was indicated in Figure 6). Our findings strongly support an important role for inhibiting the CB_1_ receptor in the pathogenesis of AMD. Topical CB_1_ blockade in the eyes, devoid of psychotropic side effects, can be considered a promising pharmacological approach for delaying or stopping the development of AMD.
